# Effect of Rapid Mold Heating on the Structure and Performance of Injection-Molded Polypropylene

**DOI:** 10.3390/polym12020341

**Published:** 2020-02-05

**Authors:** Sara Liparoti, Vito Speranza, Giuseppe Titomanlio, Roberto Pantani

**Affiliations:** 1 Department of Industrial Engineering, University of Salerno, via Giovanni Paolo II, 132-84084 Fisciano (SA), Italy; sliparoti@unisa.it (S.L.); gtitomanlio@unisa.it (G.T.); rpantani@unisa.it (R.P.); 2 Institute of Polymers, Composites and Biomaterials (IPCB), The National Research Council (Cnr), Via Previati 1/C, 23900 Lecco (LC), Italy

**Keywords:** injection molding, structure/properties relationship, cavity temperature fast modulation

## Abstract

The tailoring by the process of the properties developed in the plastic objects is the more effective way to improve the sustainability of the plastic objects. The possibility to tailor to the final use the properties developed within the molded object requires further understanding of the relationship between the properties of the plastic objects and the process conduction. One of the main process parameters that allow adjusting the properties of molded objects is the mold temperature. In this work, a thin electrical heater was located below the cavity surface in order to obtain rapid and localized surface heating/cooling cycles during the injection molding process. An isotactic polypropylene was adopted for the molding tests, during which surface temperature was modulated in terms of values and heating times. The modulation of the cavity temperature was found able to control the distribution of relevant morphological characteristics, thus, properties along the sample thickness. In particular, lamellar thickness, crystallinity distribution, and orientation were analyzed by synchrotron X-ray experiments, and the morphology and elastic modulus were characterized by atomic force microscopy acquisitions carried out with a tool for the simultaneous nanomechanical characterization. The crystalline degree slightly increased with the cavity temperature, and this induced an increase in the elastic modulus when high temperatures were adopted for the cavity surface. The cavity temperature strongly influenced the orientation distribution that, on its turn, determined the highest values of the elastic modulus found in the shear layer. Furthermore, although the sample core, not experiencing a strong flow field, was not characterized by high levels of orientation, it might show high values of the elastic modulus if temperature and time during crystallization were sufficient. In particular, if the macromolecules spent adequate time at temperatures close to the crystallization temperature, they could achieve high levels of structuring and, thus, high values of elastic modulus.

## 1. Introduction

The injection molding process is one of the most commonly used plastic formation techniques across myriad of industrial sectors, subject to the absolute ease and convenience provided by this methodology. Statistics testimony to the aforementioned fact: global injection molded plastic market size stood at 100 million tons [1]. The high automation, the short processing time, and the possibility to obtain objects with high dimensional and geometrical accuracy are the major advantages of this process [2]. Nowadays, growing interest is devoted to the increase of the sustainability of both the process and the plastic products due to the problems related to the pollution due to the high amount of plastic waste (microplastics) [3].

Generally, in order to improve the performance (mechanical, optical, etc.) of plastic products, fillers are added in the polymeric matrix. For instance, colorants, plasticizers, lubricants, antioxidants are commonly used in plastic packaging [4,5,6], and flame retardants are commonly used in plastic for electronics [7,8]. With the introduction of fillers during the process, the plastic part fulfills the requirements for the use in specific applications, but, at the same time, it reduces the sustainability of the plastic products [9]. In order to improve the sustainability of plastic products, it is important to have the possibility to tailor the properties without introducing fillers. In other words, the appropriate selection of the operating conditions must assure to tailor the properties developed in the final objects. This would increase the sustainability of the final object, making it suitable for common recycle processes [10,11].

Some attempts to tailor the properties of plastic part by the process was carried out by Turng and coworkers [12] in the production of isotactic polypropylene (iPP) films by extrusion-based processes. They found that the morphology, thus the properties, developed within the film strongly depended on the elongation degree undergone by the polymer chains during drawing. Lamberti [13] found similar behavior in the films made of iPP. Other authors proposed modification of exiting processes to tailor plastic part properties. Wang et al. [14] proposed the water-assisted injection molding for inducing the formation of fibrillar structures, which would improve the mechanical performance of the iPP parts. Also, the foaming injection molding was introduced and adopted for making lighter plastic parts adopted in the automotive field [15,16].

In the injection molding process, one of the main operating parameters that affect the properties of the final object is the mold temperature [17,18]. Surface finishing, morphology distribution, and possibility to produce micro and nano-structures on the molded surface strongly depend on the temperature adopted in the mold and, in particular, on the cavity surface [19,20,21,22,23]. The mechanical properties have been found to be dependent on the mold temperature; in particular, the tensile strength of moldings made of polyether-ether-ketone increases with the mold temperature [24]. Similar results have also been obtained in the case of polylactic acid bio-composites [25]. The impact energy and the flexural modulus have been found to depend on mold temperature for blends made of polyethylene terephthalate and polypropylene [26]. Also, the structure developed, in terms of crystallinity and amounts of crystalline phase fractions, depends on the mold temperature [27], in particular, the crystalline degree increases with mold temperature for moldings made of polypropylene.

Several methodologies can be adopted for controlling cavity surface temperature during the injection molding process, such as proximity heating [28,29], infrared heating [30], and electrical heating [31,32]. Another possibility to control the mold temperature was proposed by Minh and coworkers [33], who developed an external gas-assisted mold temperature control for improving the performance of thin rib parts. 

In this work, a thin (220 µm thickness) electrical heating device was adopted to modulate the temperature in the cavity during the injection molding process. The heating device was located just below the cavity surface in order to maximize the heating/cooling rates and to realize a localized control of the cavity temperature. Several temperature conditions, in terms of cavity surface temperature and heating times, were realized by the aforementioned devices, with the aim of tailoring plastic part structure and performance by the cavity temperature modulation. The structure developed, in terms of morphology and crystallinity distributions, and the performance, in terms of mechanical properties, within the molded objects were extensively investigated by advanced analytical techniques with the aim of correlating structure and performance to the operative conditions. In particular, the adopted techniques allowed characterizing the sample from the micrometric to nanometric level, allowing an almost direct correlation among the structures (whose dimensions extend up to tens of microns) and the mechanical performance. The thermomechanical history experienced by the polymer chains was accounted for also by means of an advanced simulation software specifically developed for the injection molding process.

## 2. Materials and Methods

### 2.1. Injection Molding Test

The injection-molded samples were obtained adopting a commercial isotactic polypropylene, T30G, supplied by Basell (Ferrara, Italy). An accurate characterization of rheology and crystallization kinetics of the resin, in both quiescent and flow conditions, is reported elsewhere [34,35,36].

The injection molding tests were performed by a Negri Bossi 70 ton machine (Negri Bossi S.p.A., Cologno Monzese, Milano, Italy) with 220 °C melt injection temperature, 2.9 cm^3^ s^−1^ average volumetric flow rate (the cavity filling time was about 0.7 s), and 25 °C mold temperature. The holding stage was performed with a pressure of 720 bar held for 6 s. A thin heating device, whose description is reported elsewhere [37], was located below the cavity surface, covering an area 12.7 mm wide and 70 mm long, realizing a localized control of the cavity surface temperature during the process. A thin steel layer (100 µm thickness) covered the heating device, protecting it from the incoming melt; thus, it was possible to state that the heating device was just below the cavity surface. The heating device adopted was able to increase the cavity surface temperature with a rate of 100 °C/s, and, being very thin (220 µm thick), it allowed a fast decrease in the temperature after deactivation. Passive tests were performed adopting the heating device as an insulant; in this case, the whole mold was left at 25 °C during the process. At least three samples for each condition were analyzed. In Table 1, the conditions of cavity surface temperatures, T, and heating times, t_h_ adopted during the injection molding experiments are reported.

**Figure 1:**
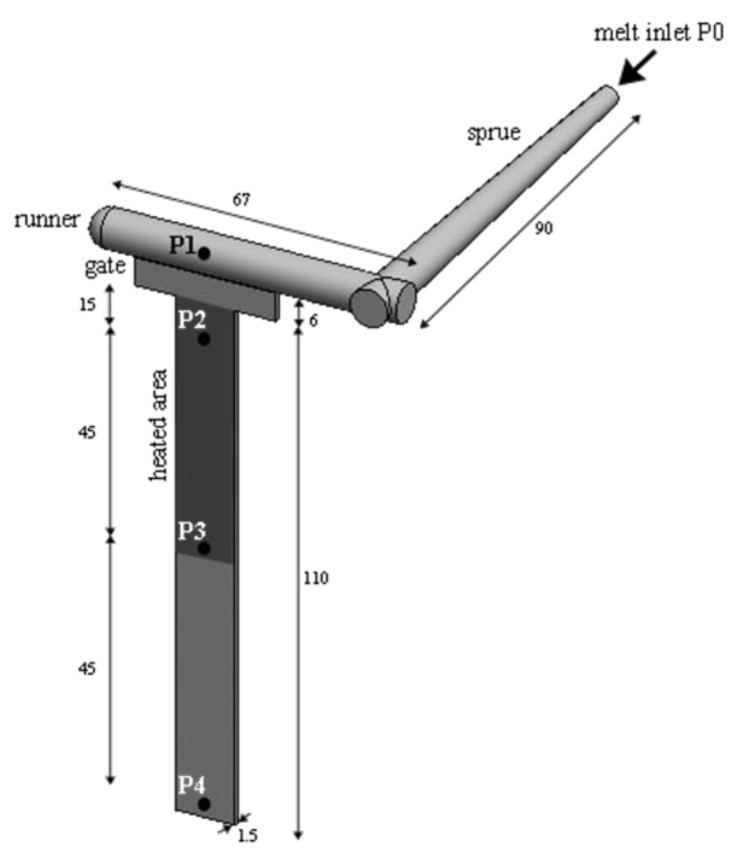
Cavity adopted for the injection molding experiments. The dimensions are referred to in millimeters. The position of each pressure transducer is also indicated along the flow direction.

Five pressure transducers (type 6171BB and 6190 BA, Kistler, Milan, Italy) were located along the flow path (namely P0, P1, P2, P3, and P4). In the position P2, a thermocouple (type T, Omega Engineering Ltd, Manchester, UK) was also located for recording the cavity surface temperature evolution. Figure 1 shows the cavity adopted for the injection molding experiments with the position of each pressure transducer along the flow path. The darker area represents the area where the heating device is located.

### 2.2. Optical Microscopy

Thin slices (100 µm) were cut along the flow-thickness plane by Leica slit microtome (mod. 625, Leica Biosystem, Buccinasco, Milan, Italy) and observed by optical microscopy in polarized light by an Olympus BX51 microscope (Olympus Italia S.R.L., Segrate, Italy) with crossed polarizer-analyzer. Optical micrographs were taken with sample slices oriented at 45° with respect to the analyzer.

### 2.3. SAXS and WAXS

The slices observed by optical microscopy were also analyzed by small and wide-angle scattering and diffraction apparatus (SAXS and WAXS, Diamond Light Source, Harwell Science and Innovation Campus, Didcot, Oxfordshire, UK) for the analyses of the crystallinity and orientation distribution along the sample thickness.

Two-dimensional WAXD patterns were circularly averaged to generate plots of diffracted intensity as a function of the azimuthal angle *q*. The plot of the diffracted intensity was analyzed by a deconvolution procedure, as described elsewhere [38]. The full spectrum was considered as a superposition of reflections due to the presence of different crystalline fractions. A total of 11 reflections were considered: 6 for the α phase (*q* = 0.99, 1.20, 1.31, 1.55, 2.03, 2.97), 1 for the mesomorphic form (*q* = 1.06), 2 for the β form (*q* = 1.14, 1.50), 1 for the γ phase (*q* = 1.44), and 1 for the amorphous halo. Each reflection was described by a Lorentzian function, and the area below each reflection, *A_i_*, was evaluated. The amount of each crystalline fraction, *ξi*, was evaluated as

(1) $$\xi i=\frac{A_{i}}{\sum_{i}^{11}A_{i}}$$

The overall crystallinity, *Xc*, was evaluated as the sum of the contribution of the crystalline fractions (α, β, γ, and mesomorphic phase).

The error of the measurement was estimated as ±3% on the percentage of each phase.

The Herman’s orientation factors for (040) and (110) planes were calculated for each spectrum. The Wilchinsky [39] equation allowed evaluating the orientation for each spectrum. The method of Wilchinsky was applied to derive the c-axis orientation using the (110)α and (040)α reflections and the angle of 72.5° between b-axis and the (110)α plane with:

(2) $$<cos^{2}\sigma >=1-0.901<cos^{2}\varphi_{040}>-1.099<cos^{2}\varphi_{110}>$$

The flow direction was taken as the reference direction. For a set of *hkl* planes, the average orientation, expressed as 〈cos^2^*φ*〉*_hkl_*, was mathematically calculated using the equation

(3) $${\langle cos^{2}\varphi \rangle }_{hkl}=\frac{\int_{0}^{\pi /2}I\left(\varphi \right)\;cos^{2}\varphi \sin\varphi }{\int_{0}^{\pi /2}I\left(\varphi \right)\sin\varphi }$$

with *φ* being the azimuthal angle, and *I (φ)* being the scattered intensity along the angle *φ*. Herman’s orientation function, *f*, was defined as

(4) $$f=\frac{3{\langle cos^{2}\varphi \rangle }_{hkl}-1}{2}$$

with *f* having a value of −0.5 with the normal of the reflection plane being perpendicular to the reference direction (*φ* = 90°), a value of 1 with the normal of the refection plane being parallel to the reference direction (*φ* = 0°), and a value of 0 with the orientation being random.

The lamellar size was also evaluated from SAXS analyses. In particular, the scattered intensity was obtained as a function of the scattering vector *q,* which is given by:

(5) $$q=\frac{4\pi }{\lambda }sen\left(\theta \right)$$

where *θ* is half of the scattering angle, and the wavelength *λ* = 1.54 Å. The measured scattering intensity could be transposed into the 1D scattering intensity using Lorentz correction. If the electron density differences in one direction are known, the average lamellar thickness *l_c_* could be obtained from [40]:

(6) $$lc=\frac{2\pi }{q_{I_{MAX}}}X_{c}$$

where $q_{I_{MAX}}$ corresponds to the maximum of the Lorentz corrected scattering intensity, and *X_c_* is the crystalline volume fraction (see Section 2.3 *SAXS and WAXS*) [41].

### 2.4. HarmoniX AFM

Specimens cut along the flow thickness plane and chemically etched, according to the procedure described in literature [42], were analyzed by atomic force microscopy equipped with HarmoniX tool (Multimode Dimension V coupled with Nanoscope V, Veeco, Santa Barbara, CA, USA) for the simultaneous characterization of the morphology and mechanical properties at the nanometrical level. In the literature, this method was found in a good agreement with data obtained from conventional mechanical analyses (namely dynamic mechanical analysis and micro–indentation) for molded produced with the iPP adopted in this work [43,44]. Tests were performed with HMX probe silicon cantilevers (Bruker, Billerica, MA, USA) with nominal radii of c.a. 10 nm. Cantilevers were calibrated using a standard polystyrene/low-density polyethylene target. The calibration of the tip was performed according to the procedure reported elsewhere [45]. The adopted vertical frequency was 44 kHz, and the torsional frequency was 1130 kHz. The cantilever vibration-free amplitude was of 750 mV, in air. The force level was modulated, adopting the amplitude setpoint. The amplitude set point was used for feedback control, as a reference value; ca. 60% of the free amplitude was selected. Imaging was performed with 0.5 Hz scan rates, considering 20 harmonics. Vertical and torsional motions were applied to the cantilever. The vertical movement allowed the acquisition of the topography; in other words, the morphology developed along the sample thickness. The torsional movement due to the tip-sample approach/withdraw generated force curves that allowed the evaluation of the elastic modulus. The Bruker NanoScope software, version 7.30, allowed the elaboration of the data in order to obtain maps of the measured properties. In particular, adhesion (force required to pull-off the tip from the surface), dissipation (area under the negative part of the force withdrawal curve) and elastic modulus maps (evaluated from force curves through the Derjaguin-Muller-Toporov model) were acquired considering a scanning area of 10 μm × 10 μm. Elastic modulus profiles along the sample thickness were obtained, using the software NanoScope Analysis version 1.80, averaging the elastic modulus distribution over the considered scanning area.

### 2.5. Simulation of the Process

A simulation software, developed at the University of Salerno for the injection molding process [19,46], was adopted for the description of the thermomechanical history undergone by the polymer.

## 3. Results

**Figure 2:**
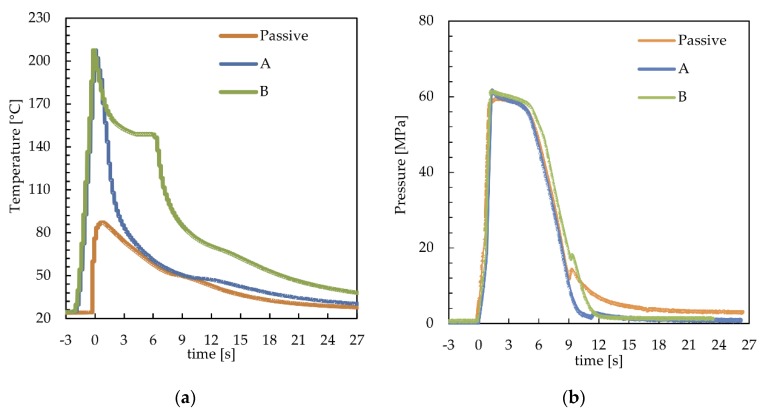
(**a**) Temperature and (**b**) pressure evolutions recorded during injection molding experiments performed with 150 °C cavity surface temperature and different heating times. The temperature and pressure evolution of the passive test were also reported.

Figure 2a shows the temperature evolutions recorded at 15 mm downstream from the gate (position P2 of Figure 1) with 150 °C cavity surface temperature and two values of the cavity surface heating time, *t_h_* = 0.7 s and 6 s (A and B). The temperature evolution recorded during the passive test was also reported for comparison. Figure 2b shows the pressure evolutions of the same tests recorded in the same position. In both graphs, the time t = 0 corresponded to the time at which the melt contacted the cavity in position P2.

For the cases A and B, a first temperature increase, from 25 °C to 150 °C (Figure 2a), was obtained activating the heating devices during the time, about 2 s, that the melt took to fill the sprue and the runner; afterward, the melt came in contact with the cavity surface, inducing an additional increase in the cavity surface temperature up to 210 °C. After the melt contacted with the cavity surface, the temperature started to decrease toward the value set for the heating device (150 °C) (kept active for 0.7 s for case A and 6 s for case B). At the heating device deactivation, the temperature started to decrease toward the temperature set for the whole mold, 25 °C. With reference to the passive case, the increase in temperature, due to the contact of the melt with the cavity surface, was significantly smaller than the cases in which the heating device was activated, also the temperature decrease rate was smaller with respect to those. In all the considered cases, the small inflection of the temperature evolutions, taking place within the temperature range 50–80 °C, was determined by the heat of crystallization that might partially overlap with the detachment of the polymer from the cavity surface (determined by the polymer shrinkage). The effect of the temperature evolution on the pressure evolution, in the same position where the temperature was recorded, is shown in Figure 2b. In the passive case, the pressure reached 23 MPa at the end of filling (0.7 s), and the increase in the cavity surface temperature induced a significant decrease of the filling pressure down to 16 MPa. For the passive and A cases, pressure evolutions in P2 after the filling stage were essentially the same, and both curves showed a change in the slope at 6 s due to the gate sealing [37]. When the cavity surface temperature was kept high for the whole holding stage (case B), the decrease in pressure delayed by some tenths of a second.

**Figure 3:**
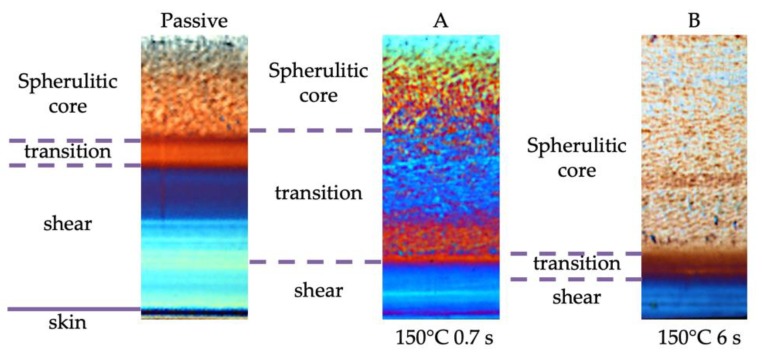
Optical micrographs of the molded samples obtained with different cavity surface temperature evolutions, from left to right: Passive, 150 °C kept for 0.7 s and 150 °C kept for 6 s (see Table 1 for the operating conditions). The slices were cut along the flow-thickness plane, and only half-thickness was shown.

The evolution of the cavity surface temperature could be adopted to modulate the morphology and properties of the molded objects. Firstly, the morphology distribution along the sample thickness was strongly influenced by the evolution of the cavity surface temperature. Figure 3 shows optical micrographs of the slices cut in position P2, along the flow thickness plane, of the samples obtained with the operating conditions reported in Table 1.

Four regions could be detected along the thickness of the passive molding sample: the skin, close to the sample surface, an oriented layer named shear, a spherulitic *core* in the inner part of the sample, and a transitional region that could be identified between the spherulitic core and the shear layer. The transitional region was characterized by a continuous transition from the fibrillar to the spherulitic morphology. The samples A and B showed regions similar to those of the passive sample, with the exception of the skin. The cavity surface temperature evolution had a significant effect on the width of each layer. When the surface temperature was kept high, the shear layer’s thickness decreased, and the transitional layer shifted toward the external part of the molding; when the heating time increased, the spherulitic core covered most of the sample thickness.

A deeper understanding of the morphology developed within the samples was achieved by the knowledge of distributions of both orientation and crystalline phase fractions within the molded samples. Both these characteristics were evaluated by WAXS analyses, performed by synchrotron acquisitions along the sample thickness of slices cut in the position P2, to which the morphologies reported in Figure 3 were referred.

**Figure 4:**
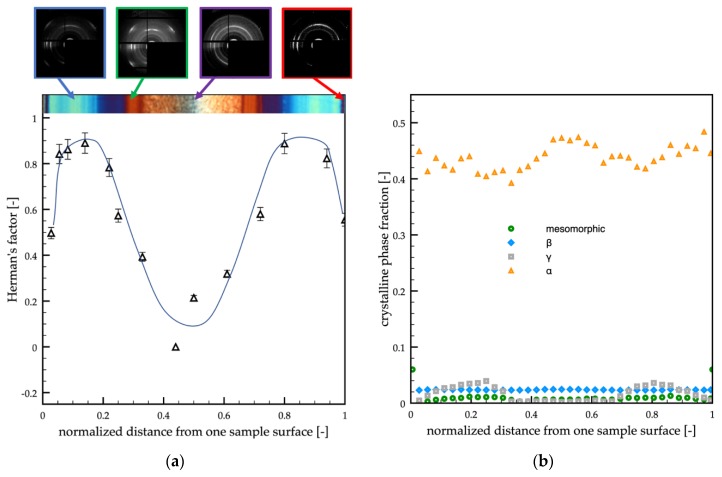
(**a**) WAXS analyses of the passive molding in different positions along the sample thickness. Each analyzed position is indicated on the optical micrograph. The distribution of the orientation in terms of Herman’s factor was also reported. (**b**) Distributions of mesomorphic, α, β, and γ-phase fractions along the sample thickness evaluated from the WAXS analyses.

Figure 4a shows the cross-section orientation distribution along the passive sample thickness. In particular, WAXS scattering images taken in different positions along the sample thickness (as shown on the topmost of the optical micrograph) and Herman’s factors (as obtained from WAXS scattering images) were reported versus the normalized distance (normalized with respect to the whole sample thickness) from one sample surface.

The WAXS analyses showed that the orientation distribution, in terms of Herman’s factor (see Figure 4a), was characterized by two maxima, located in the center of the shear layer regions (*d* = 0.18 and *d* = 0.82), by a minimum in the inner part of the sample and intermediate values at the sample surface. Figure 4b shows the crystalline phase fraction distributions (mesomorphic phase, α-phase, β-phase, and γ-phase) along the normalized distance from one sample surface. As expected for the adopted grade of iPP, the α-phase was the predominant phase, whereas only small fractions of other phases were detected. Interestingly, the distribution of the γ-phase was quite similar to the distribution of the orientation. This finding was consistent with the fact that the high orientation and high pressure induce the formation of the γ-phase [47,48]. Small fractions of the mesomorphic phase were concentrated at the sample surfaces. The β-phase fraction was found negligible over the whole sample thickness.

**Figure 5:**
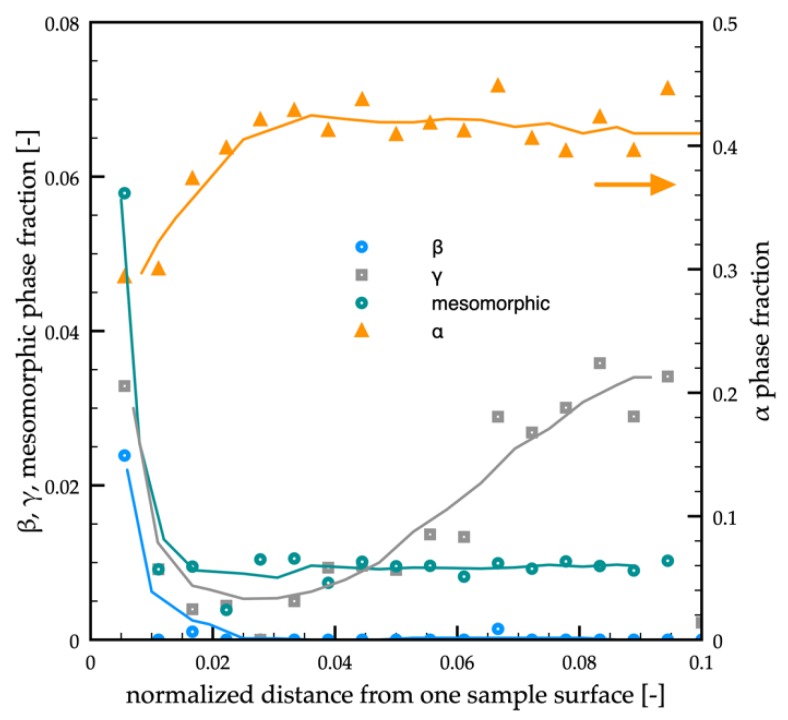
Distribution of mesomorphic, α, β, and γ-phases fractions evaluated from the WAXS analyses for the passive sample, up to 0.1 normalized distance from one sample surface.

Figure 5 shows the distribution of mesomorphic, α, β, and γ-phases fractions evaluated from the WAXS analyses for the passive sample up to 0.1 normalized distance from one sample surface.

The α-phase fraction increased from 0.30 to 0.45 in the interval 10–100 μm from sample surface (0.005–0.05 normalized distance). The mesomorphic phase fraction reached 0.06 in the first 10 µm from the sample surface, and it decreased down to 0.01 toward the inner parts of the sample (within the first 50 μm).

**Figure 6:**
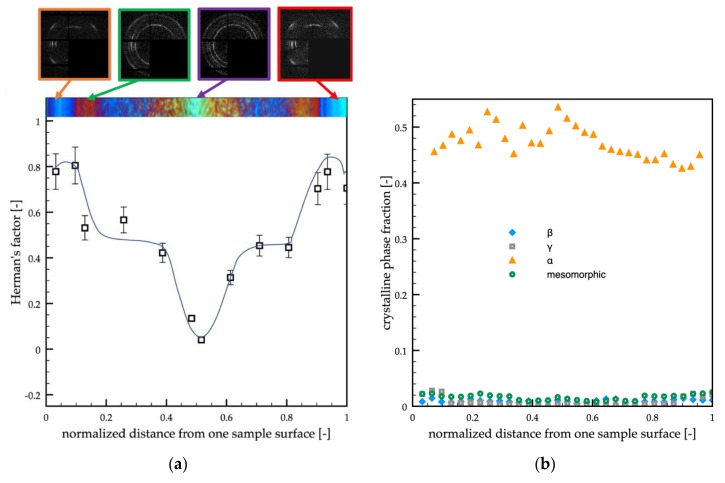
(**a**) WAXS analyses of the A molding in different positions along the sample thickness. Each analyzed position is indicated on the optical micrograph. The distribution of the orientation in terms of Herman’s factor was also reported. (**b**) Distributions of mesomorphic, α, β, and γ-phase fractions along the sample thickness evaluated from the WAXS analyses

Similar analyses were performed on the samples obtained with a 150 °C cavity surface temperature, and 0.7 s heating time (case A in Table 1). Figure 6 shows the orientation distribution (in terms of Herman’s factor) along the sample thickness, the WAXS analyses in different positions along the sample thickness, and the optical micrograph of the sample along the flow thickness plane.

The orientation of sample A was smaller with respect to the passive case. The maxima of orientation were located close to the sample surface, and the minimum was located in the sample core. In the regions between the core and the shear layer, the orientation was found almost constant and slightly lower with respect to the passive case. The distribution of the crystalline phase fractions (Figure 6b) showed again that the α-phase was the predominant one, only a small fraction of γ-phase was present, and it was mainly located close to the sample surface. The mesomorphic and the β-phase fractions were found negligible (smaller than 0.02).

**Figure 7:**
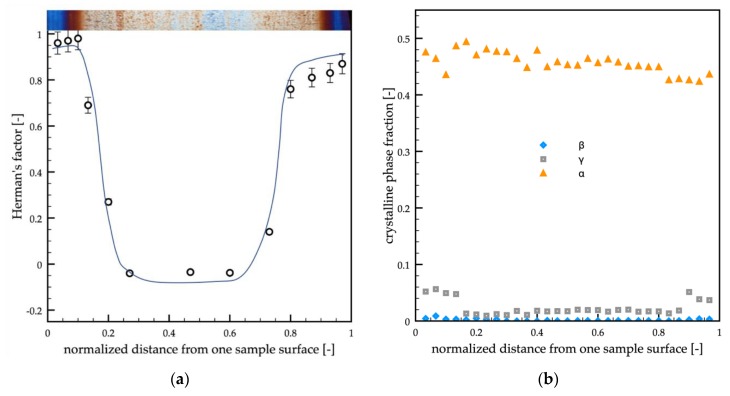
(**a**) WAXS analyses of the B molding in different positions along the sample thickness. Each analyzed position is indicated on the optical micrograph. The distribution of the orientation in terms of Herman’s factor was also reported. (**b**) Distributions of α, β, and γ-phase fractions along the sample thickness evaluated from the WAXS analyses.

Similar analyses were performed on the samples obtained with a 150 °C cavity surface temperature and 6 s heating time (case B, in Table 1). Figure 7 shows the orientation distribution (Herman’s factor) and the crystalline phase fraction distributions along the sample thickness (Figure 7a,b, respectively). The maxima of orientation were located in very narrow regions close to the sample surface, and, differently from the passive sample, the reduction of the orientation at the sample surface was not observed. It is possible to observe a significant decrease in Herman’s factor in the thin transition region. In the wide spherulitic core, the orientation was found essentially constant and comparable with the minimum values observed in the passive and A samples. The distribution of the crystalline phase fractions (Figure 7b) showed again that the predominant phase was the α-phase, only a small fraction of γ-phase was found and mainly located close to the sample surface, whereas neither the mesomorphic nor the β-phase were detected in a significant amount.

## 4. Elastic Modulus Distributions

**Figure 8:**
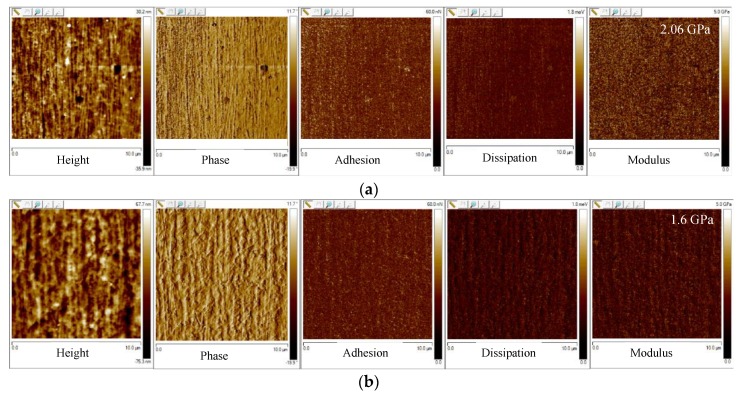
AFM maps of the height, phase, adhesion, dissipation, and elastic modulus, for the passive sample, acquired along the sample thickness at different distances from the surface: (**a**) 150 µm, namely the shear layer; (**b**) 300 µm, namely the transition layer.

In order to assess the influence of the morphology and the crystalline structure on the properties of the molded samples, nanomechanical tests were performed. Figure 8 shows the AFM maps acquired on the passive sample along the thickness at two different distances from the sample surface: 150 μm (in the shear layer), 300 μm (in the transitional layer). Five maps were shown for each distance: the height that allowed analyzing the morphology of the sample, the phase, the adhesion, the dissipation, and the elastic modulus maps.

The height maps showed the morphology of the passive sample in different areas along the thickness direction. At 150 µm distance from the surface, fibrillar morphology could be detected; at a higher distance from the sample surface (namely 300 μm), the distance between the fibrils increased, and the fibrils appeared to be thicker.

The phase-contrast microscopy, using AFM, is generally adopted to detect changes in composition or viscoelastic properties of polymers [49]. A viscoelastic material would strain periodically when subjected to periodic stress, such as the stress experienced during the AFM analysis in tapping mode. However, the strain response lagged the stress by a phase angle that is characteristic of the material. The presence of the regions with different values of phase lag could be attributed to the presence of regions characterized by elastic/viscoelastic behavior. The viscoelastic behavior could be attributed to the presence of parts with small crystalline fractions. This was confirmed by dissipation maps. The dissipation was the energy that was dissipated by the AFM tip during the withdraw from the sample. The dissipated energy and the adhesion were higher in the regions with a higher amount of viscous-viscoelastic material. Obviously, the elastic modulus was higher in the regions where the viscoelastic part was lower, i.e., in the shear layer.

**Figure 9:**
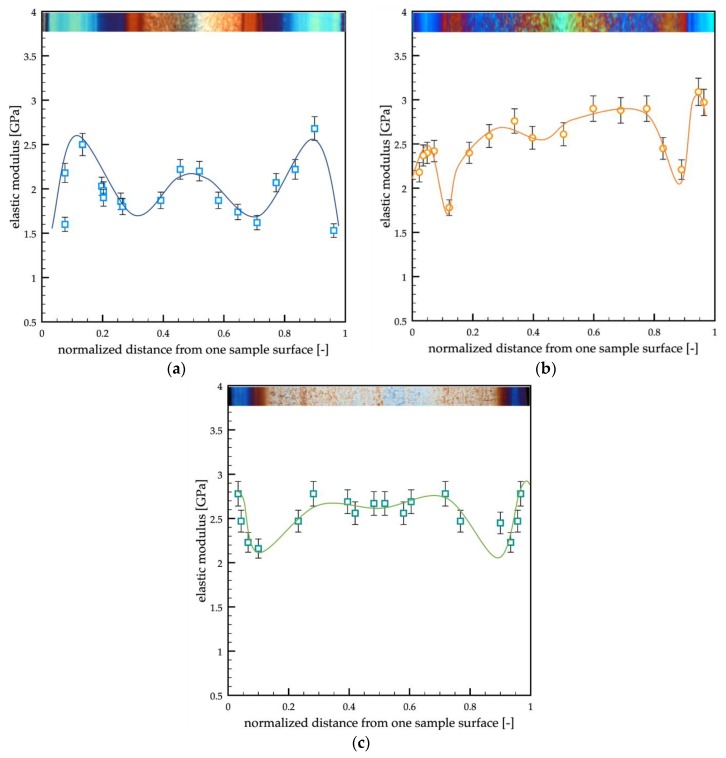
Elastic modulus distributions measured with the HarmoniX technique along the thickness of the (**a**) passive, (**b**) A, and (**c**) B sample.

Figure 9 shows the elastic modulus distributions obtained for the samples passive, A, and B.

The passive sample showed two maxima of the elastic modulus in the shear regions, the lowest values could be detected at the sample surface and in the transition regions, and the core was characterized by intermediate values of the elastic modulus. The samples A and B showed similar trends of the elastic modulus distribution. Furthermore, they were characterized by higher values of the elastic modulus with respect to the passive sample. The high values of the elastic modulus characteristic of the shear region moved toward the sample surface, accordingly with the reduction of the shear layer thickness due to the increase in the cavity surface temperature.

## 5. Discussion

**Figure 10:**
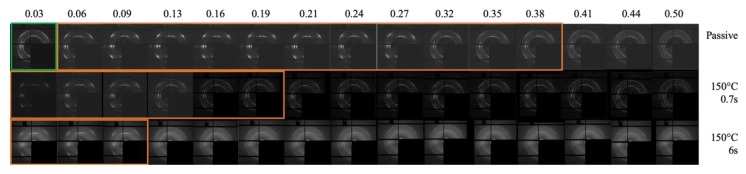
WAXS scattering distribution of the passive, A, and B moldings at different distances from one sample surface (the normalized distance is reported in the topmost of the figure). The higher orientation regions are delimited by orange borders, whereas the region with poor orientation (close to the surface of the passive sample) is delimited with the green borders.

Figure 10 shows the WAXS analyses at different distances from the sample surface for the passive, A, and B cases. The orange borders delimit the regions with a high orientation level.

The diffraction scattering intensity distribution consisted of five diffraction rings associated with different lattice planes of iPP, namely (110), (040), (130), (111), and (−131), from inner to outer circles, which were typical of the α-phase. An additional (300) lattice plane appeared in the transition layer, corresponding to the reflection of β-phase. γ-phase (117) diffraction ring was also observed. It was possible to detect traces of β-phase in sample A, where the β-phase ring was detectable up to 200 µm distance from the sample surface. The formation mechanism of β-phase was related to the shear flow, which could induce the formation of α-row structures. The surface of these row structures provided nucleation sites for the β-phase. The portion of β-phase depended on process conditions: the β-phase formed at low shear rates and low cooling rates, and its content in the moldings considered in this work was negligible. At a high shear rate, strong shear flow induced highly oriented structures, i.e., shish-kebabs, formed by α-phase [50,51,52]. The γ-phase ring was visible in the passive sample and in the sample A up to 450 µm from the sample surface. The γ-phase found in the regions close to the sample surface could be attributed to both the intense flow and to the fact that in these positions, the material experienced solidification under high pressure. The WAXS distribution showed on the left corner of the first line of Figure 10, highlighted with green color, was related to the skin region close to the surface of the passive sample; in this region, the mesomorphic phase fraction achieved 6%, whereas the mesomorphic phase fraction is negligible in the inner part of the sample. The observed distribution of the mesomorphic phase is consistent with the distribution reported in the literature [53]. The formation of the mesomorphic phase took place as a consequence of fast cooling experienced by the melt when contacted the cold surface of the mold [41,54,55]. As a further confirmation that the formation of the mesomorphic phase occurred under fast cooling conditions, the samples A and B, obtained with 150 °C cavity surface temperature, showed only a negligible fraction (smaller than 2%) of the mesomorphic phase since, in those conditions, the cooling rate was smaller than in the passive case.

**Figure 11:**
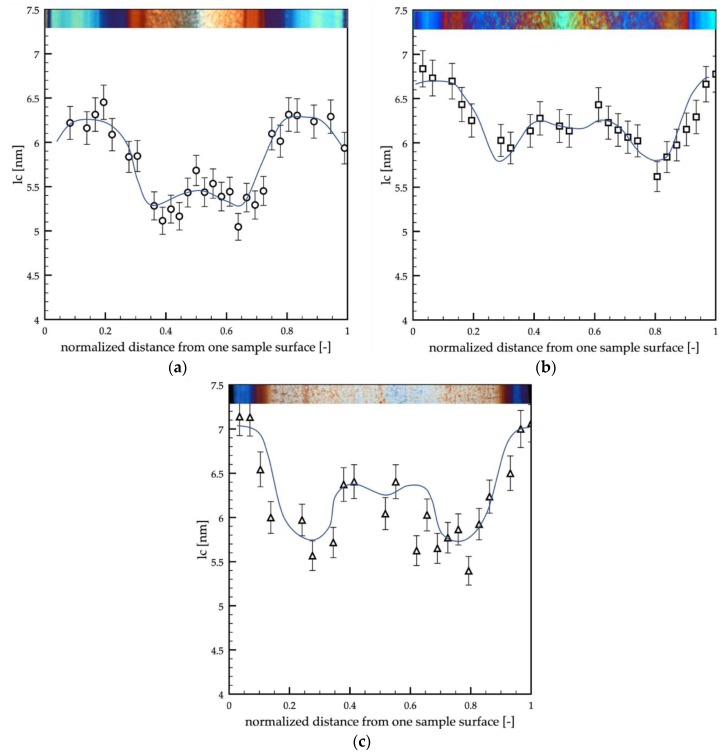
Lamellar size distributions along the thickness of the passive (**a**), A (**b**), and B (**c**) samples.

The lamellar size distributions, *lc*, were evaluated from the SAXS analyses, and their results are shown in Figure 11. The information about the lamellar thickness allows understanding the structure developed at a smaller level with respect to the observed morphology (spherulites or fibrils).

In the three cases analyzed in this work, the lamellar size, *lc*, was found to range between 5 and 7 nm. The passive sample showed slightly smaller values of the lamellar size with respect to the other two cases. In all the cases, *lc* was found larger at the sample surface, namely in the shear layers, and the values were almost constant in these regions. The width of the area in which the lamellar size was constant depended on the evolution of temperature on the cavity surface. In particular, the width of areas decreased with the increase in surface temperature and heating time. The transition regions were characterized by the smallest values of the lamellar size, whereas the core showed intermediate values.

The shear rate at the surface oriented (and stretched) the polymer chains, thus determining an entropy decrease within the system. Consequently, in the shear layer, the material crystallized at higher temperatures, with respect to the quiescent condition, and the formation of thicker lamellae was favored [56,57,58,59]. Sample B (obtained with high cavity surface temperature, 150 °C, kept for a long time, 6 s) showed a slight increase in the lamellar thickness, *lc,* especially in the core region, with respect to the passive and A samples. It seemed that the higher temperature, 150 °C, kept for a long time, 6 s, during the process, enhanced the structuring of the polymer chains, determining the increase of lamellar thicknesses.

**Figure 12:**
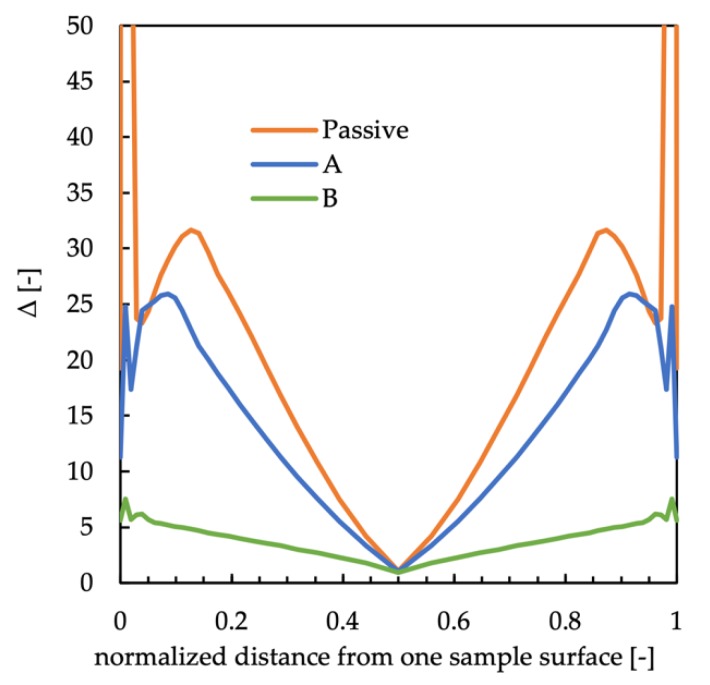
Distributions of the stretch, Δ, calculated by the simulation at the end of the process for the passive case and for cases A and B.

A first attempt to find a relationship between structure and properties, especially mechanical property, was done by comparing elastic modulus distributions with the data obtained by SAXS and WAXS analyses for each molding condition. The WAXS analyses showed that the distributions of total crystallinity fractions were essentially homogeneous along the sample thickness, and the α-phase was the predominant crystalline phase, whereas only small fractions of the other phases were detectable. Furthermore, the increase in the cavity surface temperature induced a small increase in the crystallinity degree. Although small, the increase in the crystallinity degree could induce a small increase in the elastic modulus. Furthermore, the distributions of orientation, also obtained by WAXS analyses, appeared to be similar to the elastic modulus distribution. In particular, the maxima of the elastic modulus and orientation occurred at the same distance from the sample surface. The orientation was correlated with the stretch distribution that could be evaluated by the simulation software developed at the University of Salerno. Figure 12 shows the stretch distributions along the sample thickness calculated at the end of the process.

The stretch distribution of the passive sample was characterized by the presence of a maximum in the shear layer; beyond the shear layer, the stretch decreased down to the minimum in the core. The regions characterized by these values of stretch were composed of tightly packed fibrils. In the regions where the stretch decreased, a transitional layer characterized by a continuous transition from the fibrils to the spherulites was found. The formation of these areas was due to weak flows and slower cooling rates with respect to shear layers. The stretch distributions of the samples A and B are also shown in Figure 12. The stretch distribution of samples A and B showed a behavior similar to the one showed by the passive sample, with a maximum of the stretch in the shear layer. However, since the shear layer moved toward the sample surface, the maximum of the stretch was located closer to the sample surface with respect to the maximum of the passive sample. Sample B showed the lowest values of the stretch along the whole thickness according to the increase in the cavity surface temperature and heating time duration. These results confirmed the hypothesis previously introduced that the formation of the transitional areas was due to the weak flow, meaning small values of the stretch and slow cooling rates.

It seemed that in the shear layer, the stretch behavior was the main responsible for the high values of the elastic modulus. At the sample surface, even if the stretch assumed the highest value, the fast cooling rate, undergone by the molecules, prevented the molecular structuring, and thus lowest values of elastic modulus were found. The increase in the cavity surface temperature and heating time duration induced a decrease in the width of the regions with higher values of stretch and, thus, elastic modulus.

**Figure 13:**
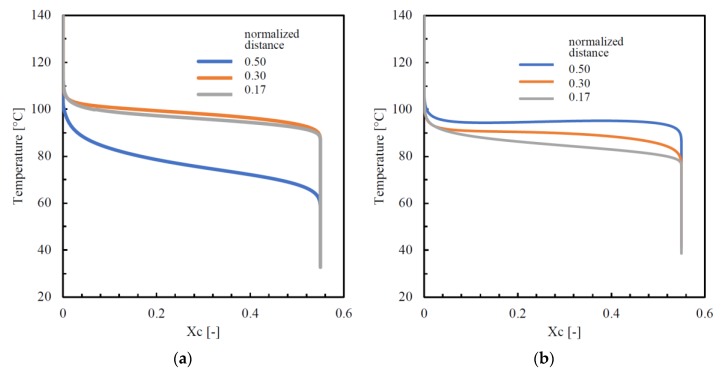
The temperature during the crystallization for the samples passive (**a**) and B (**b**).

In the core region, elastic modulus distributions seemed to be not correlated to the orientation and stretch profiles. Indeed, in the core region, orientation and stretch assumed minimum values in all the considered cases, whereas the elastic modulus showed intermediate values. It seemed that the elastic modulus followed the behavior of the lamellar thickness that was correlated to the structuring during crystallization. Figure 13a,b shows the temperature evolution during the crystallization for two samples, passive and sample B, at several dimensionless distances from the sample surface, evaluated by the simulation software for injection molding developed at the University of Salerno.

Figure 13a shows that, at the two distances (0.17 and 0.30 normalized distances) from the sample surface, the evolutions of the temperature during crystallization were very close, although the distances from the surfaces were different, this was because:

1. for given crystallization kinetics, crystallization temperature increased when cooling rate decreased (which happened as the distance from the surface increased);

2. for a given cooling rate, crystallization temperature decreased when the shear rate (and thus the molecular stretch) decreased (which happened as the distance from the surface increased).

As the distance from the surface increased, the effect of a decrease in cooling rate on the crystallization temperature was partially compensated by the decrease in shear rate, and thus crystallization took place at about the same temperature in the two positions 0.17 and 0.30.

In the core region, the crystallization started at lower temperatures, and the temperature kept on decreasing, from 100 °C to 60 °C, while the crystallization proceeded. Such a large difference in behavior with respect to the regions close to the sample surface was due to the fact that the crystallization in the core occurred after flow cessation, namely during the cooling, whereas in the regions close to the sample surface, the crystallization occurred during the filling and the first instants of the packing. Vice versa, when the temperature of the cavity surface was kept constant and high, at 150 °C, during filling and packing (test B, Figure 13b), most of the molecular stretch relaxed over the whole cross-section before crystallization and, consequently, the differences in crystallization temperature with the distances from the surface (0.17, 0.3, and 0.5) were essentially due to differences in the cooling rates. Thus, the sequence of the curves in Figure 13b followed the sequence of cooling rates, which decreased as the distance from the surface increased. Minor effects might be due to the residues of molecular stretch not yet completely relaxed. The evolution of temperature during crystallization was determined by a balance between the local cooling rate and the rate of heat generation due to the crystallization rate. In the core zone (the blue curve in Figure 13b), the crystallization temperature remained essentially constant and high; there (being far from the surface), the heat was lost under a slow rate, and the heat generation could slowly prevail, determining a small temperature increase. Thus, the time the polymer remained in the high range of the crystallization temperature, and available for structuring, was long in the whole cross-section, especially in the central zone. In previous work, the increase in the elastic modulus was attributed to the possibility of the polymer chains of structuring [44]. It appears that the longer is the time a molecule spends within the crystallization temperature range, the higher would be the structuring level and, therefore, the higher would be the elastic modulus. Obviously, this effect overlaps the effect of the molecular stretch. The higher values of the modulus in the core for the passive sample and the high values of the modulus along the whole thickness of sample B were consistent with the previous statements.

Summarizing, the increase in the elastic modulus in the core with respect to the transition areas was due to a decrease in the cooling rate that, on its turn, allowed better structuring of the molecules. The increase in the structuring level allowed an increase in the elastic modulus in the central part of the sample, which was responsible for the mechanical behavior of the whole sample.

## 6. Conclusions

Injection-molded samples of isotactic polypropylene were produced, modulating cavity surface temperature during the process, with the aim of tailoring the performance by the process. The samples were deeply characterized by adopting several techniques. Final properties distributions along sample thickness were obtained, adopting polarized optical microscopy, SAXS, WAXS, and HarmoniX atomic force microscopy. Crystallinity degree was found essentially constant along the sample thickness and close to 45% for all the analyzed samples; only a slightly increase of the crystallinity degree was found on increasing the cavity surface temperature. The α-phase was the predominant phase. Low fractions of mesomorphic phase were found only close to the sample surface, where it reached 6% for the sample obtained in conventional injection molding conditions, whereas, in the samples obtained with the cavity surface temperature modulation, the mesomorphic fractions were found to be negligible. The γ-phase fraction was found only in the shear layer, where it reached values smaller than 10%. The γ-phase fraction distribution was found quite similar to the distribution of the orientation. The β-phase fraction was found negligible for all the analyzed samples.

The morphology developed within the samples was accurately characterized by both optical microscopy and atomic force microscopy. Four areas were detected along the sample thickness: a skin, characterized by poorly oriented and structured features, a shear layer, a spherulitic core, and a transition layer between the shear and the spherulitic layers. The shear layer was composed of tightly packed fibrils. This area was characterized by the highest values of the orientation; Herman’s factor was close to 1 for all the tests reported in this work. The elastic modulus found in the shear layer was the highest one, from 2.5 to 3.0 GPa, and it slightly increased with the cavity surface temperature and the heating time. The transition layer was characterized by fibrils that gradually evolve toward spherulitic structures. The orientation in this area gradually decreased from the values of the shear layer to the values of the spherulitic core. The elastic modulus showed the minimum values, from 1.7 to 2.2 GPa, in the transition. These values were close to the values found in the skin, 1.6 GPa. The spherulitic core was characterized by isotropic structures, the spherulites, which showed the smaller values of orientation, and Herman’s factor was close to zero. The values of the elastic modulus in the spherulitic core were found to be intermediate between the values found in the shear and the values found in the transition layer. Interestingly, the values of the elastic modulus in the spherulitic core depended on the modulation of the cavity temperature; in particular, the higher were the cavity surface temperature and the heating time, the higher was the elastic modulus. The lamellar thickness, which was also evaluated from the SAXS analyses, showed distributions similar to the elastic modulus distribution. The maximum values (6.5–7.0 nm) were found in the shear, and the minimum values (5.0–5.5 nm) were found in the transition. The core showed intermediate values of lamellar thickness; furthermore, the lamellar thickness increased in the core with the cavity temperature and heating time.

The distributions of the mechanical properties and orientation were compared with the stretch the polymer chains experienced during the process, which was evaluated by the software developed at the University of Salerno for the injection molding process. In the shear and transitional layers, elastic modulus distribution showed a behavior similar to the orientation and stretch distributions. In the core layer, high values of elastic modulus seemed to be related to the increase in lamellar size. The interpretation of the aforementioned observation was given on the basis of the temperature evolution during the material crystallization and calculated using the aforementioned simulation software. In the core layer, even if the molecules did not experience high levels of orientation/stretch, they spent a long time within the crystallization temperature range, reaching a higher level of structuring and thus high values of elastic modulus. The skin layer, although characterized by the maximum level of stretch, showed small values of the elastic modulus. In this area, indeed, the fast cooling limited the structuring. Therefore, distributions of elastic modulus along the sample thickness seemed to be correctly interpreted by combining stretch and level of structuring.

## Figures and Tables

**Table 1:** Operating conditions adopted during the injection molding experiments Passive, A and B (T = temperature at the cavity surface; t_h_ = cavity surface heating time).

Test Name	T Cavity Surface (°C)	t_h_ (s)
Passive	25	-
A	150	0.7
B	150	6

## References

[1] Saipriya I. Injection Molded Plastic Market Growth. Top Four Trends Driving Injection Molded Plastic Market over 2016–2023: U.S. to Remain a Prominent Revenue Contributor. https://gminsights.wordpress.com/2017/08/03/injection-molded-plastic-market/.

[2] Greener J., Wimberger-Friedl R. (2006). Precision Injection Molding.

[3] He D., Luo Y., Lu S., Liu M., Song Y., Lei L. (2018). Microplastics in soils: Analytical methods, pollution characteristics and ecological risks. TrAC Trends Anal. Chem..

[4] Hahladakis J.N., Velis C.A., Weber R., Iacovidou E., Purnell P. (2018). An overview of chemical additives present in plastics: Migration, release, fate and environmental impact during their use, disposal and recycling. J. Hazard. Mater..

[5] Cherif Lahimer M., Ayed N., Horriche J., Belgaied S. (2017). Characterization of plastic packaging additives: Food contact, stability and toxicity. Arab. J. Chem..

[6] Magalhães da Silva S.P., Lima P.S., Oliveira J.M. (2016). Non-isothermal crystallization kinetics of cork-polymer composites for injection molding. J. Appl. Polym. Sci..

[7] Schneider D., Hübner C., Bourbigot S. (2019). New approach for the efficient attainment of flame retardancy using multi component injection molding. AIP Conf. Proc..

[8] Furio A., Landi G., Altavilla C., Sofia D., Iannace S., Sorrentino A., Neitzert H.C. (2017). Light irradiation tuning of surface wettability, optical, and electric properties of graphene oxide thin films. Nanotechnology.

[9] Eriksen M.K., Pivnenko K., Olsson M.E., Astrup T.F. (2018). Contamination in plastic recycling: Influence of metals on the quality of reprocessed plastic. Waste Manag..

[10] Al-Salem S.M., Lettieri P., Baeyens J. (2009). Recycling and recovery routes of plastic solid waste (PSW): A review. Waste Manag..

[11] Badia J.D., Ribes-Greus A. (2016). Mechanical recycling of polylactide, upgrading trends and combination of valorization techniques. Eur. Polym. J..

[12] Zhou Y.-G., Turng L.-S., Shen C.-Y. (2010). Morphological evolution and orientation development of stretched iPP films: Influence of draw ratio. J. Polym. Sci. Part B Polym. Phys..

[13] Lamberti G. (2011). Flow-induced crystallization during isotactic polypropylene film casting. Polym. Eng. Sci..

[14] Wang B., Huang H.-X., Wang Z.-Y. (2015). Process-induced phase and crystal morphologies in water-assisted injection molded polypropylene/polymeric β-nucleating agent blend parts. Polym. Eng. Sci..

[15] Ameli A., Kazemi Y., Wang S., Park C.B., Pötschke P. (2017). Process-microstructure-electrical conductivity relationships in injection-molded polypropylene/carbon nanotube nanocomposite foams. Compos. Part A Appl. Sci. Manuf..

[16] Sun X., Kharbas H., Peng J., Turng L.S. (2015). A novel method of producing lightweight microcellular injection molded parts with improved ductility and toughness. Polymer (Guildf).

[17] Guilong W., Guoqun Z., Huiping L., Yanjin G. (2010). Analysis of thermal cycling efficiency and optimal design of heating/cooling systems for rapid heat cycle injection molding process. Mater. Des..

[18] Huang C.T., Hsien I.S., Tsai C.H., Chiou Y.C., Tang C.C. (2011). The effects of various variotherm processes and their mechanisms on injection molding. Int. Polym. Process..

[19] Liparoti S., Speranza V., Pantani R., Titomanlio G. (2019). Process Induced Morphology Development of Isotactic Polypropylene on the Basis of Molecular Stretch and Mechanical Work Evolutions. Materials (Basel).

[20] Liparoti S., Speranza V., Pantani R. (2018). Replication of micro- and nanofeatures in injection molding of two PLA grades with rapid surface-temperature modulation. Materials (Basel).

[21] Weng C., Wang F., Zhou M., Yang D., Jiang B. (2018). Fabrication of hierarchical polymer surfaces with superhydrophobicity by injection molding from nature and function-oriented design. Appl. Surf. Sci..

[22] Baruffi F., Gülçür M., Calaon M., Romano J.-M., Penchev P., Dimov S., Whiteside B., Tosello G. (2019). Correlating nano-scale surface replication accuracy and cavity temperature in micro-injection moulding using in-line process control and high-speed thermal imaging. J. Manuf. Process..

[23] Jiang J., Wang S., Sun B., Ma S., Zhang J., Li Q., Hu G.-H. (2015). Effect of mold temperature on the structures and mechanical properties of micro-injection molded polypropylene. Mater. Des..

[24] Park K., Kim Y.-S. (2009). Effect of Mold Temperature on Mechanical Properties of an Injection-Molded Part with Microfeatures. J. Polym. Eng..

[25] Nagarajan V., Zhang K., Misra M., Mohanty A.K. (2015). Overcoming the Fundamental Challenges in Improving the Impact Strength and Crystallinity of PLA Biocomposites: Influence of Nucleating Agent and Mold Temperature. ACS Appl. Mater. Interfaces.

[26] Kuzmanović M., Delva L., Cardon L., Ragaert K. (2016). The Effect of Injection Molding Temperature on the Morphology and Mechanical Properties of PP/PET Blends and Microfibrillar Composites. Polymers (Basel).

[27] Wang W., Zhao G., Guan Y., Wu X., Hui Y. (2015). Effect of rapid heating cycle injection mold temperature on crystal structures, morphology of polypropylene and surface quality of plastic parts. J. Polym. Res..

[28] Shih S.-Y., Nian S.-C., Huang M.-S. (2016). Comparison between single- and multiple-zone induction heating of largely curved mold surfaces. Int. Commun. Heat Mass Transf..

[29] Chen S.-C., Minh P.S., Chang J.-A., Huang S.-W., Huang C.-H. (2012). Mold temperature control using high-frequency proximity effect induced heating. Int. Commun. Heat Mass Transf..

[30] Gao S., Qiu Z., Ma Z., Yang Y. (2017). Development of high efficiency infrared-heating-assisted micro-injection molding for fabricating micro-needle array. Int. J. Adv. Manuf. Technol..

[31] De Meo A., De Santis F., Pantani R. (2018). Dynamic local temperature control in micro-injection molding: Effects on poly(lactic acid) morphology. Polym. Eng. Sci..

[32] Xiao C.-L., Huang H.-X., Yang X. (2016). Development and application of rapid thermal cycling molding with electric heating for improving surface quality of microcellular injection molded parts. Appl. Therm. Eng..

[33] The Nhan P., Do T.T., Anh Son T., Son Minh P. (2019). Study on External Gas-Assisted Mold Temperature Control for Improving the Melt Flow Length of Thin Rib Products in the Injection Molding Process. Adv. Polym. Technol..

[34] Pantani R., Nappo V., De Santis F., Titomanlio G. (2014). Fibrillar morphology in shear-induced crystallization of polypropylene. Macromol. Mater. Eng..

[35] Pantani R., Speranza V., Titomanlio G. (2015). Simultaneous morphological and rheological measurements on polypropylene: Effect of crystallinity on viscoelastic parameters. J. Rheol. (N. Y. N. Y)..

[36] De Santis F., Pantani R., Titomanlio G. (2016). Effect of shear flow on spherulitic growth and nucleation rates of polypropylene. Polymer (Guildf).

[37] Liparoti S., Sorrentino A., Titomanlio G. (2018). Temperature and pressure evolution in fast heat cycle injection molding. Mater. Manuf. Process..

[38] Murthy N.S., Minor H. (1990). General procedure for evaluating amorphous scattering and crystallinity from X-ray diffraction scans of semicrystalline polymers. Polymer (Guildf).

[39] Wilchinsky Z.W. (1963). Orientation in cold-rolled polypropylene. J. Appl. Polym. Sci..

[40] Caelers H.J.M., Govaert L.E., Peters G.W.M. (2016). The prediction of mechanical performance of isotactic polypropylene on the basis of processing conditions. Polymer (Guildf).

[41] Liparoti S., Sorrentino A., Guzman G., Cakmak M., Titomanlio G. (2015). Fast mold surface temperature evolution: Relevance of asymmetric surface heating for morphology of iPP molded samples. RSC Adv..

[42] White H.M., Bassett D.C. (1998). On Row Structures, Secondary Nucleation and Continuity in Alpha- Polypropylene. Polymer (Guildf).

[43] Liparoti S., Sorrentino A., Speranza V., Titomanlio G. (2017). Multiscale mechanical characterization of iPP injection molded samples. Eur. Polym. J..

[44] Liparoti S., Sorrentino A., Speranza V. (2017). Micromechanical Characterization of Complex Polypropylene Morphologies by HarmoniX AFM. Int. J. Polym. Sci..

[45] Liparoti S., Speranza V., Sorrentino A., Titomanlio G. (2017). Mechanical Properties Distribution within Polypropylene Injection Molded Samples: Effect of Mold Temperature under Uneven Thermal Conditions. Polymers (Basel).

[46] Pantani R., Speranza V., Titomanlio G. (2017). Effect of flow-induced crystallization on the distribution of spherulite dimensions along cross section of injection molded parts. Eur. Polym. J..

[47] Kalay G., Zhong Z., Allan P., Bevis M.J. (1996). The occurrence of the γ-phase in injection moulded polypropylene in relation to the processing conditions. Polymer (Guildf).

[48] Anczykowski B., Gotsmann B., Fuchs H., Cleveland J.P., Elings V.B. (1999). How to measure energy dissipation in dynamic mode atomic force microscopy. Appl. Surf. Sci..

[49] Scott W. (2003). Use of phase imaging in atomic force microscopy for measurement of viscoelastic contrast in polymer nanocomposites and molecularly thick lubricant films. Ultramicroscopy.

[50] Speranza V., Liparoti S., Pantani R., Titomanlio G. (2018). Hierarchical structure of iPP during injection molding process with fast mold temperature evolution. Mater. Rev..

[51] Mi D., Xia C., Jin M., Wang F., Shen K., Zhang J. (2016). Quantification of the Effect of Shish-Kebab Structure on the Mechanical Properties of Polypropylene Samples by Controlling Shear Layer Thickness. Macromolecules.

[52] Wang K., Chen F., Zhang Q., Fu Q. (2008). Shish-kebab of polyolefin by “melt manipulation” strategy in injection-molding: A convenience pathway from fundament to application. Polymer (Guildf).

[53] Pantani R., Coccorullo I., Speranza V., Titomanlio G. (2005). Modeling of morphology evolution in the injection molding process of thermoplastic polymers. Prog. Polym. Sci..

[54] De Rosa C., Auriemma F., Galotto N.G., Di Girolamo R. (2012). Mesomorphic form of isotactic polypropylene in stereodefective polypropylene: Solid mesophase or liquid-crystal like structure. Polymer (Guildf).

[55] Liparoti S., Titomanlio G., Sorrentino A. (2016). Analysis of asymmetric morphology evolutions in iPP molded samples induced by uneven temperature field. AIChE J..

[56] Housmans J.-W., Gahleitner M., Peters G.W.M., Meijer H.E.H. (2009). Structure–property relations in molded, nucleated isotactic polypropylene. Polymer (Guildf).

[57] Zhong G.-J., Li Z.-M., Li L.-B., Mendes E. (2007). Crystalline morphology of isotactic polypropylene (iPP) in injection molded poly(ethylene terephthalate) (PET)/iPP microfibrillar blends. Polymer (Guildf).

[58] Marand H., Xu J., Srinivas S. (1998). Determination of the Equilibrium Melting Temperature of Polymer Crystals: Linear and Nonlinear Hoffman−Weeks Extrapolations. Macromolecules.

[59] Ryan A.J., Stanford J.L., Bras W., Nye T.M.W. (1997). A synchrotron X-ray study of melting and recrystallization in isotactic polypropylene. Polymer (Guildf).

